# Dr. Guernsey’s Defense

**Published:** 1890-07

**Authors:** Egbert Guernsey


### DR. GUERNSEY'S DEFENSE.

Editor of The Homœopathic Physician:—Your favor of the 31st of May, calling attention to a marked copy of the June issue of The Homœopathic Physician which you kindly sent, is at hand. On turning to the page indicated in your note, I find a letter from Dr. E. Carleton. The resolution to which Dr. Carleton refers is identical in sentiment, if not entirely in language, with the one passed by the New York County Homœopathic Medical Society several years ago, but at a later date rescinded by a small majority under the leadership of Dr. Bayard. The commissioners had sent the communication from the County Society respecting the Ward's Island Hospital to the Medical Board to answer. The resolution was part of the proceedings of the meeting, and was forwarded to the commissioners as part of the minutes. Several weeks later, Dr. Carleton, who was not present at the meeting—in fact, he has not been present at any of the regular meetings of the Medical

Board for several years—addressed me the letter published in your journal. Simply as a matter of courtesy, this letter was read to the Board and a minute made of Dr. Carleton's dissent. No answer was sent, as in my estimation none was required. It is a pity that Dr. Carleton has not always been so eager to avoid the appearance of consent by silence. At a later date, I was requested by several members of the Board to get the opinion of its members in regard to a change of name, as the commissioners contemplated changing the names of several of the institutions under their care. This I did, in a letter addressed to each member of the Board. I have no doubt that Dr. Carleton thought it was obligatory upon him as a gentleman to send to the public print a letter written by the president of a hospital in the strict discharge of his official duty.

Possibly, if Dr. Carleton would take more direct interest in the hospital itself and attend more closely to his official duties, it might save him the trouble of visiting his supposed grievances upon the public.

Respectfully yours,

EGBERT GUERNSEY.

